# Determinants of Glycan Receptor Specificity of H2N2 Influenza A Virus Hemagglutinin

**DOI:** 10.1371/journal.pone.0013768

**Published:** 2010-10-29

**Authors:** Karthik Viswanathan, Xiaoying Koh, Aarthi Chandrasekaran, Claudia Pappas, Rahul Raman, Aravind Srinivasan, Zachary Shriver, Terrence M. Tumpey, Ram Sasisekharan

**Affiliations:** 1 Harvard-MIT Division of Health Sciences and Technology, Singapore-MIT Alliance for Research and Technology, Department of Biological Engineering, Koch Institute for Integrative Cancer Research, Massachusetts Institute of Technology (MIT), Cambridge, Massachusetts, United States of America; 2 Influenza Division, Centers for Disease Control and Prevention (CDC), Atlanta, Georgia, United States of America; Hallym University, Republic of Korea

## Abstract

The H2N2 subtype of influenza A virus was responsible for the Asian pandemic of 1957-58. However, unlike other subtypes that have caused pandemics such as H1N1 and H3N2, which continue to circulate among humans, H2N2 stopped circulating in the human population in 1968. Strains of H2 subtype still continue to circulate in birds and occasionally pigs and could be reintroduced into the human population through antigenic drift or shift. Such an event is a potential global health concern because of the waning population immunity to H2 hemagglutinin (HA). The first step in such a cross-species transmission and human adaptation of influenza A virus is the ability for its surface glycoprotein HA to bind to glycan receptors expressed in the human upper respiratory epithelia. Recent structural and biochemical studies have focused on understanding the glycan receptor binding specificity of the 1957-58 pandemic H2N2 HA. However, there has been considerable HA sequence divergence in the recent avian-adapted H2 strains from the pandemic H2N2 strain. Using a combination of structural modeling, quantitative glycan binding and human respiratory tissue binding methods, we systematically identify mutations in the HA from a recent avian-adapted H2N2 strain (A/Chicken/PA/2004) that make its quantitative glycan receptor binding affinity (defined using an apparent binding constant) comparable to that of a prototypic pandemic H2N2 (A/Albany/6/58) HA.

## Introduction

The 20th century witnessed three influenza pandemics: the Spanish flu of 1918 (H1N1), the Asian flu of 1957-58 (H2N2) and the Hong Kong flu of 1967-68 (H3N2). Among these subtypes the H1N1 and H3N2 continue to circulate in the human population leading to epidemic outbreaks annually and the H1N1 subtype was responsible for the 2009 ‘swine flu’ pandemic (2009 H1N1). The H2N2 subtype had stopped circulating in humans by 1968, however H2 subtype viruses are occasionally isolated from swine and avian species [1], [2], [3]. The circulation of avian H2 strains in domestic birds and pigs increase the risk of human exposure to these viruses and reintroduction of the viruses to the human population. Such a reintroduction will pose a significant global health threat given the lack of pre-existing immunity in a huge subset of the human population born after 1968.

One of the main steps in the evolution of a pandemic influenza virus is the acquisition of genetic changes that enable it to adapt to the human host in order to replicate efficiently and transmit rapidly resulting in widespread and sustained disease in humans [4], [5], [6]. The critical first step in the host infection by the virus is the binding of the viral surface glycoprotein hemagglutinin (HA) to sialylated glycan receptors, complex glycans terminated by *N*-acetylneuraminic acid (Neu5Ac) expressed on the host cell surface [7], [8], [9]. Glycans terminating in Neu5Ac that is α2→6-linked to the penultimate sugar are predominantly expressed in human upper respiratory epithelia [10], [11], [12] and serve as receptors for human-adapted influenza A viruses (henceforth referred to as *human receptors*). On the other hand, glycans terminating in Neu5Ac that is α2→3 -linked to the penultimate sugar residue, serve as receptors for the avian-adapted influenza viruses (henceforth referred to as *avian receptors*) [13].

The molecular interactions of HA with avian and human receptors have been captured using a topology-based definition of glycan receptors [10], [14]. Glycan array platforms comprised of representative avian and human receptors have been widely employed to study the glycan receptor binding of HAs and whole viruses [15], [16], [17], [18]. The relative binding affinities of recombinantly expressed HAs from avian- (such as H1N1 and H5N1) and human-adapted (such as H1N1 and H3N2) viruses to avian and human receptors have been quantified by analyzing these HAs (or whole viruses) in a dose-dependent manner on glycan array platforms [10], [19], [20], [21]. Furthermore, the glycan array binding properties of the HAs have been shown to correlate with their binding to physiological glycan-receptors in human respiratory tissues [10], [19], [21]. Importantly, it has been shown that the human receptor-binding affinity of H1N1 HAs correlated with the efficiency of airborne viral transmission in the ferret animal model [19], [21], which is an established model to evaluate viral transmissibility in humans [22], [23], [24], [25], [26]. Such a relationship has yet to be shown for the H2N2 subtype.

Previous structural and biochemical studies have provided insights into interactions of the receptor binding site (RBS) of HA with avian and human receptors for both wild type (*WT*) and mutant forms of HA derived from the 1957-58 H2N2 pandemic strains [27], [28]. However, it has been recently demonstrated that changes in the interactions between amino acids within and proximal to the RBS, arising from substitutions due to antigenic drift or reassortment, have profound effects on HA-glycan interactions which in turn influences the glycan binding affinity of HA [19], [20]. This observation is particularly relevant to HA from recent avian-H2 strains that have diverged considerably in sequence compared to the HA sequence of the pandemic H2N2 strains [29]. Therefore in order to monitor changes in the recent avian H2-subtype viruses that would possibly lead to their human-adaptation, it is important to understand the mutations in their HA that would confer human receptor-binding affinity that is quantitatively in the same range as that of HA from the 1957-58 human-adapted H2N2 pandemic viruses.

In this study, we have systematically analyzed the effects of mutations in the glycan RBS of pandemic and recent avian H2N2 HAs on their respective glycan-binding specificities. The HA from a representative 1957-58 pandemic H2N2 strain, A/Albany/6/58 (*Alb58*), was chosen as a reference human-adapted HA. The HA from a representative avian H2N2 virus, A/Chicken/Pennsylvania/2004 (*CkPA04)*, which is among the most recent strains isolated from birds was also evaluated in this study [29]. We first characterized the glycan receptor-binding affinity and human respiratory tissue binding properties of these avian- and human-adapted H2N2 HAs. The glycan receptor-binding affinity of HA is quantitatively defined using an apparent binding constant *K_d_'* that takes into account the cooperativity and avidity in the multivalent HA-glycan interactions as described previously [21]. Next, using homology-based structural models of *Alb58* HA-human receptor and *CkPA04* HA-avian receptor complexes we analyzed the RBS of these HAs and designed and evaluated mutations in *CkPA04* HA that would make its human receptor binding affinity in the same range as that of *Alb58* HA.

## Results

### Characterization of glycan receptor- binding specificity of Alb58 HA

**Figure 1:**
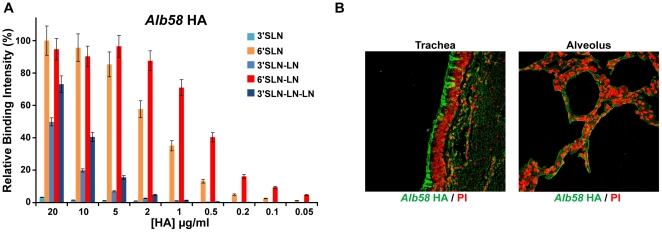
Glycan receptor-binding specificity of *Alb58* HA. ***A***, shows dose-dependent direct glycan array binding of *Alb58* HA which shows high affinity binding to human receptors in comparison with avian receptor binding. ***B***, shows extensive staining of apical surface of human tracheal epithelia and observable staining of alveolar tissue section by *Alb58* HA (in *green*) shown against propidium idodide staining (in *red*).

We have previously developed a dose-dependent glycan array binding assay [10], [21] to quantitatively characterize glycan receptor binding affinity of HA by calculating an apparent binding constant *K_d_'*. *Alb58* HA was recombinantly expressed and analyzed using this assay. *Alb58* HA bound with high affinity to the representative human receptors, 6′SLN (*K_d_'* ∼35 pM) and 6′SLN-LN (*K_d_'* ∼5 pM) (**Figure 1A**). Notably, the binding affinity of *Alb58* HA to 6′SLN-LN is in the same range as that of the pandemic H1N1 (A/South Carolina/1/1918 or SC18) HA [21]. However unlike SC18 HA, *Alb58* HA also showed substantial binding to the representative avian receptors 3′SLN-LN (*K_d_′* ∼1.5 nM) and 3′SLN-LN-LN (*K_d_'* ∼1 nM) on the glycan array (**Figure 1A**). Staining of *Alb58* HA on human upper respiratory tracheal tissue sections revealed extensive binding of the protein to the apical side (**Figure 1B**) and thus correlated with its high affinity binding to human receptors. Additionally, the substantial α2→3 sialylated glycan binding of *Alb58* observed in the glycan array assay was also reflected in its binding to the human deep lung alveolar tissue (**Figure 1B**) that predominantly expresses these glycans [10], [21].

Previous studies have pointed to the roles played by the amino acids in positions 226 and 228 in the RBS of H2N2 HAs in governing the glycan receptor binding specificity [27], [28]. The observation includes the fact that HA from most human H2N2 isolates has Leu226 and Ser228 within its RBS, whereas HA from most avian H2 isolates has Gln226 and Gly228. To understand the roles of these residues on the quantitative glycan receptor binding affinity of *Alb58* HA, three mutant forms of *Alb58* were designed. Two of these mutants possessed a single amino acid change, Leu226→Gln (*Alb58-QS* mutant) and Ser228→Gly (*Alb58-LG*). The third mutant carried two amino acid changes, Leu226→Gln er228→Gly (*Alb58-QG*).

**Figure 2:**
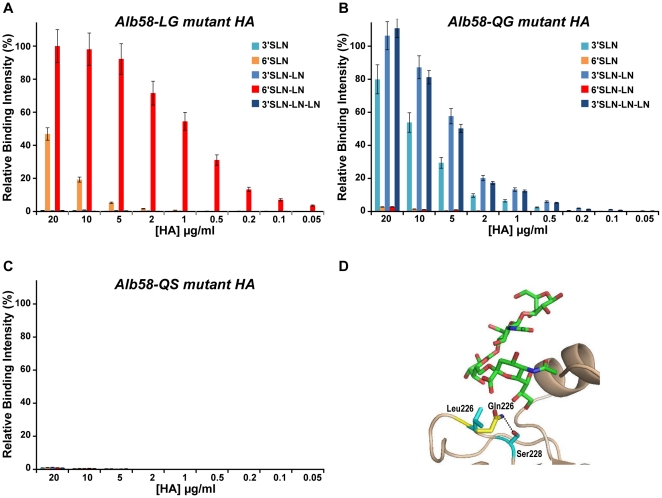
Glycan receptor-binding specificity of mutant forms of *Alb58* HA. Shown in ***A-C*** is the dose-dependent glycan array binding of *Alb58-LG*, *Alb58-QG* and *Alb58-QS* mutants respectively. A single amino acid change from Ser228→Gly (*Alb58-LG* mutant) leads to a loss of avian receptor binding observed in *Alb58* HA. An additional Leu226→Gln mutation (on *Alb58-LG*) completely transforms the binding specificity by making the *Alb58-QG* mutant bind predominantly to avian receptors. *Alb58-QS* mutant shows loss of both avian and human receptor binding. ***D*** shows homology based structural model of *Alb58-QS* mutant (RBS part is shown as a cartoon in *beige*) with the human receptor. Both the Leu226 and Gln226 side chains are marked. The Gln226 in the mutant is positioned to interact with Ser228 hence making the 226 position less favorable for contacts with both human and avian receptors.

*Alb58-LG* mutant retained the human receptor binding specificity of the *WT Alb58* HA but showed a complete loss in the avian receptor binding in the dose-dependent direct binding assay (**Figure 2A**). On the other hand, *Alb58-QG* mutant showed a complete loss in human receptor binding and but displayed a substantial binding to avian receptors in contrast to *Alb58* HA (**Figure 2B**). Surprisingly, *Alb58-QS* mutant exhibited little to no binding to either the avian or human glycan receptor (**Figure 2C**). Circular dichroism analysis of *Alb58-QS* ruled out the possibility of *Alb58-QS* being misfolded (*data not shown*). A homology-based structural model of the *Alb58-QS* mutant was constructed to investigate the molecular basis of the observed biochemical binding property. Analysis of the glycan receptor-binding site of this mutant in the model showed that Ser228 is positioned to form a hydrogen bond with Gln226 (**Figure 2D**). The interaction between Gln226 and Ser228 potentially disrupts the favorable positioning of Gln226 for optimal contact with avian receptor. This observation offers an explanation for the loss of avian receptor binding in the *Alb58-QS* mutant. Furthermore, the absence of contacts between Gln226 and human receptor could explain the loss of human receptor binding.

### Mutations in RBS of CkPA04 and their effects on its glycan receptor binding specificity

**Figure 3:**
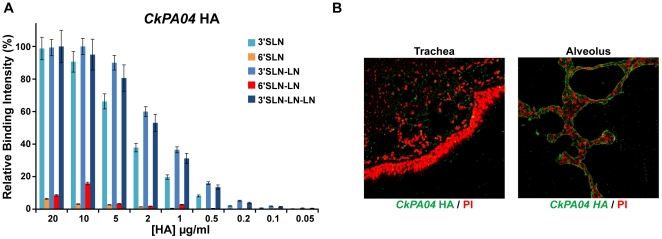
Glycan receptor-binding specificity of *CkPA04* HA. ***A***, shows dose-dependent direct glycan array binding of *CkPA04* HA which shows high affinity binding to avian receptors in comparison with human receptors. ***B***, shows extensive alveolar staining and minimal staining of apical surface of the human tracheal epithelia by *CkPA04* HA (in *green*) shown against propidium idodide staining (in *red*).

The dose-dependent glycan array binding of *CkPA04* HA showed high affinity binding to the representative avian receptors 3′SLN, 3′SLN-LN and 3′SLN-LN-LN with minimal binding to human receptors (**Figure 3A**). Furthermore, the glycan array binding property of *CkPA04* correlated with its extensive binding to the human alveolar tissues and minimal binding to the apical side of the tracheal tissues (**Figure 3B**).

**Figure 4:**
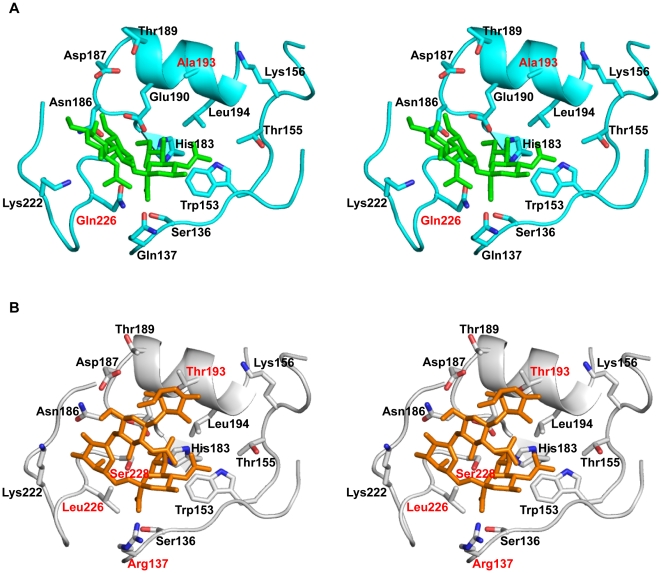
Homology-based structural model of HA-glycan receptor complexes. ***A***, stereo view of the RBS (shown as cartoon in *cyan*) of *CkPA04 HA* – avian receptor structural complex constructed using co-crystal structure of A/Chicken/NY/91-avian receptor (PRB ID: 2WR2) as a template. The resolved coordinates of the avian receptor (Neu5Acα2→3Galβ1→3GlcNAc) are shown using a stick representation (in *green*). ***B***, stereo view of RBS (shown as cartoon in *gray*) of *Alb58 HA* – human receptor complex constructed using co-crystal structure of A/Singapore/1/57– human receptor (PDB ID: 2WR7) as the template. The resolved coordinates of the human receptor (Neu5Acα2→6Galβ1→4GlcNAcβ1→3Gal) are shown using a stick representation (in *orange*). The side chains of the key residues involved in interaction with glycan receptor are shown and labeled. The residues in the RBS that differ between *CkPA04* and *Alb58* HA are labeled in *red*.

To understand the molecular aspects of the H2 HA-glycan receptor interaction, we constructed homology-based structural models of the *CkPA04*-avian (**Figure 4A**) and the *Alb58*-human receptor complexes (**Figure 4B**). Based on these structural models of *CkPA04* and *Alb58* HAs, the amino acids positioned to interact with the glycan receptors were compared (**Table 1**). In addition to the differences in 226 and 228 positions, there were differences in other positions including 137 and 193. The amino acids at positions 137 and 193 are oriented to interact with Neu5Acα2→6Gal motif as well as sugars beyond this motif in the context of the human receptor (and potentially play a role in antigenic variations among current strains of H2 viruses; see discussion). These differences potentially impinge on the human receptor binding of H2N2 HA. Notably, *CkPA04* HA differs from earlier avian-adapted H2N2 HAs in the 137 and 193 positions. Therefore, while the Gln226→Leu and Gly228→Ser substitutions would make the RBS of earlier avian-adapted H2N2 HAs almost identical to that of the pandemic *Alb58* HA, additional amino acid changes are required in the more recent avian-adapted HAs, including *CkPA04*.

**Table 1:** Comparison of key amino acids in the RBS of *CkPA04* and *Alb58* Has.

	136	137	153	155	156	183	186	187	189	190	193	194	222	226	228
*CkPA04*	S	*Q*	W	T	K	H	N	D	T	E	*A*	L	K	*Q*	*G*
*Alb58*	S	*R*	W	T	K	H	N	D	T	E	*T*	L	K	*L*	*S*

Based on the above analysis, three sets of mutations were progressively made on *CkPA04* to improve its contacts with the human receptor. The first mutant comprised of the two amino acid change Gln226→Leu/Gly228→Ser (*CkPA04-LS*). The second mutant, *CkPA04-TLS*, included an additional Ala193→Thr amino acid change in the *CkPA04-LS* HA. The third mutant, *CkPA04-RTLS*, was generated by introducing an additional Gln137→Arg mutation in the *CkPA04-TLS* HA. These HA mutants were recombinantly expressed and characterized in terms of their quantitative glycan receptor binding affinity and human tissue binding properties.

**Figure 5:**
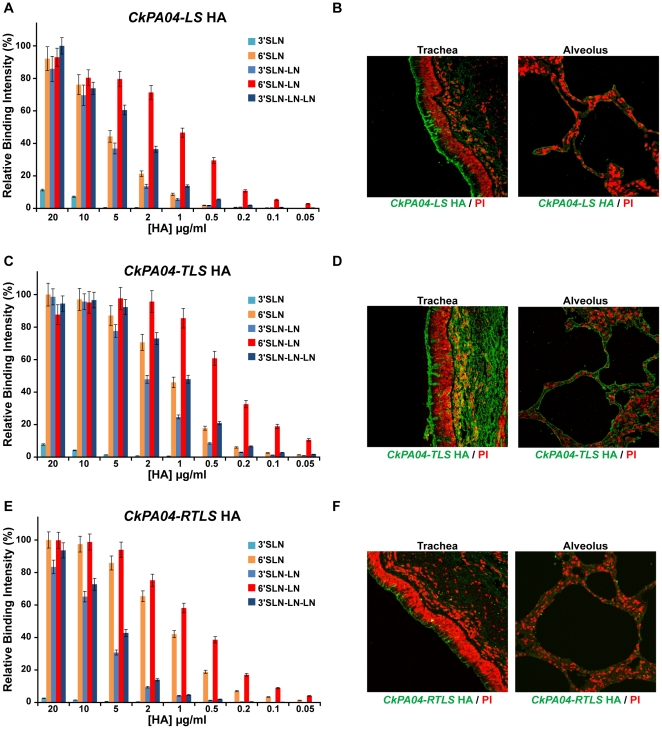
Glycan receptor-binding specificity of mutant forms of *CkPA04* HA. Shown in the figure is the dose-dependent glycan receptor binding (***A, C, E***) and human tissue binding (***B, D, F***) of *CkPA04-LS*, *CkPA04-TLS* and *CkPA04-RTLS* mutants respectively. All the mutants show substantial improvement in the human receptor binding and reduction in avian receptor binding in comparison to the *WT CkPA04 HA* as observed in both the glycan array tissue-binding experiments.

**Figure 6:**
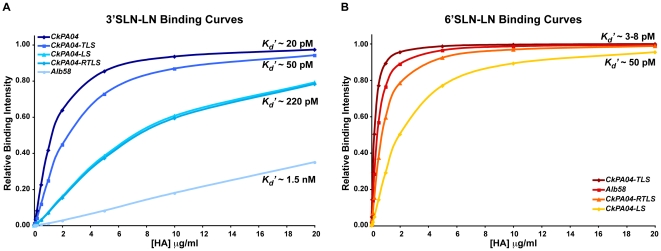
Glycan receptor-binding affinities of the mutant forms of *CkPA04* HA. ***A***, shows the theoretical binding curves (with the apparent binding constant *K_d_'*) that depict the differences in the binding affinity of the *WT* and mutant H2N2 HAs to the representative avian receptor (3′SLN-LN). ***B***, shows the theoretical binding curves that depict the differences in the binding affinity of the *WT* and mutant H2N2 HAs to the representative human receptor (6′SLN-LN). The range of *K_d_'* values (3–8 pM) is shown for *CkPA04-TLS*, *Alb58* and *CkPA04-RTLS* that is contrasted with the *K_d_'* value of *CkPA04-LS*. The binding curves were generated by fitting to the Hill equation (see Methods) and plotting the theoretically calculated fractional saturation (*y-axis*) against HA concentration (*x-axis*). The *n* value for all the binding events is around 1.3.

*CkPA04-LS* showed decreased binding to avian receptors and substantial binding to human receptors in comparison with *CkPA04* (**Figure 5A**). *CkPA04-TLS* showed substantially higher binding signals to both human and avian receptors when compared to *CkPA04-LS* (**Figure 5C**). *CkPA04-RTLS* on the other hand showed increased binding signals to human receptor and similar binding signals to avian receptor as compared to *CkPA04-LS* (**Figure 5E**). The human respiratory tissue binding of these mutant H2 HAs was in agreement with their observed glycan array binding (**Figure 5B, 5D, 5F**). The dose-dependant glycan binding data of the described HAs were used to calculate *K_d_'* and *n* values (*n* ∼1.3 for all the HAs) by fitting the binding data to the Hill equation (for multivalent binding) and this was then used to generate theoretical binding curves to clearly distinguish the relative binding affinities of *WT* and mutant H2 HAs to representative avian and human receptors (**Figure 6**). The human receptor binding affinity of *CkPA04-LS* (*K_d_'* ∼50 pM) was 10-fold lower than that of the *Alb58* HA (*K_d_'* ∼5 pM). On the other hand the human receptor binding affinity of both *CkPA04-TLS* (*K_d_'* ∼3 pM) and *CkPA04-RTLS* (*K_d_'* ∼8 pM) were several fold higher than that of *CkPA04-LS* and in the same range as that of *Alb58* HA. The avian receptor binding affinity of *CkPA04-TLS* (*K_d_'* ∼50 pM) was in the same range as that of the *WT CkPA04* HA (*K_d_'* ∼20 pM) and several fold higher than that of *CkPA04-LS* (*K_d_'* ∼220 pM) and *CkPA04-RTLS* (*K_d_'* ∼220 pM). Therefore, among the different mutants, *CkPA04-RTLS* was the closest to *Alb58* HA in terms of its relative human to avian receptor binding affinity. Based on our structural understanding, this observation is consistent with the fact that the RBS of *CkPA04-RTLS* and *Alb58* were very similar to each other, including extended range contacts with the glycan receptor beyond the Neu5Ac linkage.

## Discussion

Our study highlights the importance of integrating a systematic sequence and structure analysis of HA-glycan molecular interactions and a quantitative binding assay to study the effects of these interactions on the biochemical glycan receptor binding affinity of HA.

Previous studies have focused on amino acid substitutions in 226 and 228 positions in the RBS of pandemic H2N2 HAs [28]. Recently the glycan receptor-binding properties of the *Alb58* virus and the *WT* and mutant forms (with substitutions in 226 and 228 positions in HA) of a related pandemic H2N2 virus – A/El Salvador/2/57 (or *ElSalv57*) were characterized by analyzing these whole viruses in a dose dependent fashion on the glycan array platform [30]. The glycan receptor-binding properties of the recombinant *Alb58* HA reported in the present study are in good agreement with those obtained using the whole viruses [30]. Our results further augment these observations by characterizing the effect of substitutions in the 226 and 228 position on the quantitative glycan receptor binding affinity of *Alb58* HA.

In addition to the previously noted 226 and 228 positions [27], [28], our systematic sequence and structural analysis of H2 HA-glycan complexes revealed key differences between *CkPA04* and *Alb58* HAs in other positions, such as 137 and 193. By progressively designing mutations in *CkPA04* we demonstrate that substitutions at the 137 and 193 positions (in addition to those in 226 and 228 positions) considerably alter the glycan receptor binding affinity. In fact, introducing these additional amino acid changes (*CkPA04-TLS* and *CkPA04-RTLS* mutants) leads to a 10-fold increase in the human receptor binding affinity compared to that of the *CkPA04-LS* mutant and makes the affinity in the range of that observed for the pandemic H2N2 HA (*Alb58*). Therefore, monitoring the mutations in these additional positions in the RBS is important for understanding changes in glycan receptor binding affinity of the H2 HAs. Moreover, these additional positions are also a part of antigenic loops and hence are likely to undergo constant substitutions as a result of antigenic drift in the H2 viruses to escape antibody neutralization. Monitoring these mutations also have important implications in vaccine development should a scenario arise wherein recent avian or swine H2 viruses are able to gain a foothold in the human population.

The apparent binding constant *K_d_'* calculated in our study is used primarily to compare the relative binding affinities of different recombinant HAs by taking into account a defined spatial arrangement of HA (that is fixed for all the HAs) relative to the glycans. Among the various factors that influence the efficient viral transmissibility in humans we have shown in both the 1918 pandemic H1N1 and the recently declared 2009 pandemic H1N1 that the binding affinity to the human receptors (quantified using *K_d_'*) correlates with the transmissibility of the virus via respiratory droplets in ferrets [19], [21]. The human receptor binding affinity of *Alb58* HA being in the same range as that of the SC18 HA taken together with the efficient respiratory droplet transmission of the *Alb58* virus [30] extends this correlation to the H2N2 viruses. Furthermore, given that *Alb58* virus transmits efficiently via respiratory droplets in ferrets, our results underscores the fact that a complete switch from avian to human receptor binding is not the critical determinant for human adaptation of influenza A virus HAs. Both the quantitative glycan array binding and human tissue binding results of *Alb58* HA show substantial avian receptor binding. Instead, it appears that the high affinity binding to human receptors is a common factor shared by H2 HA with that of other human-adapted virus subtypes (H1 and H3) [10], [21] and therefore this property appears to be a necessary determinant for efficient human adaptation and transmission. In summary our studies offer important strategies to monitor the evolution of human-adaptive mutations in the HA of currently circulating avian H2 influenza A viruses.

## Materials and Methods

### Homology based modeling of *CkPA04* HA- and *Alb58* HA-glycan structural complexes

The co-crystal structures of A/Singapore/1/57 H2N2 HA – human receptor (PDB ID: 2WR7) and A/ck/NewYork/91– avian receptor (PDB ID: 2WR2) were used as templates to model the structural complexes of *Alb58*– human receptor and *CkPA04*– avian receptor respectively. Homology modeling was performed using the SWISS-MODEL web-based program (URL: http://swissmodel.expasy.org/SWISS-MODEL.html).

### Cloning, mutagenesis and expression of HA

The *Alb58* and *CkPA04* plasmids were gifts from Dr. Terrence Tumpey and Dr. Adolfo Garcia-Sastre respectively. The human and avian *WT* H2N2 HA genes were subcloned into a pAcGP67A vector to generate pAcGp67-*Alb58*-HA and pAcGp67-*CkPA04*-HA respectively for baculovirus expression in insect cells. Using pAcGp67-*CkPA04*-HA as a template the gene was mutated to yield pAcGp67-LS-HA [Gln226Leu, Gly228Ser], pAcGp67-TLS-HA [Ala193Thr, Gln226Leu, Gly228Ser] and pAcGp67-RTLS-HA [Gln137Arg, Ala193Thr, Gln226Leu, Gly228Ser]. The primers for mutagenesis were designed using PrimerX (http://bioinformatics.org/primerx/) and synthesized by IDT DNA technologies (Coralville, IA). The mutagenesis reaction was carried out using the QuikChange Multi Site-Directed Mutagenesis Kit (Stratagene, CA) *Alb58*, *CkPA04*, *CkPA04-LS*, *CkPA04-TLS* and *CkPA04-RTLS* baculoviruses were created from their respective plasmids, using Baculogold system (BD Biosciences, CA) as per the manufacturer's instructions. The baculoviruses were used to infect 300 ml suspension cultures of Sf9 cells (Invitrogen, Carlsbad, CA) cultured in Sf-900 II SFM medium (Invitrogen, Carlsbad, CA). The infected cultures were monitored and harvested 4–5 days post-infection. The soluble trimeric form of HA was purified from the supernatant of infected cells using modification of the protocol described previously [31]. In brief, the supernatant was concentrated using Centricon Plus-70 centrifugal filters (Millipore, Billerica, MA) and the trimeric HA was recovered from the concentrated cell supernatant using affinity chromatography with columns packed with Ni-NTA beads (Qiagen, Valencia, CA). The fractions containing HA were pooled together and subjected to ultrafiltration using Amicon Ultra 100 K NMWL membrane filters (Millipore, Billerica, MA). The protein was reconstituted in PBS and concentrated. The purified protein concentration was determined using Bio-Rad's protein assay (Bio-Rad, CA).

### Dose dependent direct glycan array-binding assay

To investigate the multivalent HA-glycan interactions a streptavidin plate array comprising representative biotinylated α2→3 and α2→6 sialylated glycans as described previously [21]. The glycans 3′SLN, 3′SLN-LN, 3′SLN-LN-LN are representative avian receptors. 6′SLN and 6′SLN-LN are representative human receptors. LN corresponds to lactosamine (Galβ1-4GlcNAc) and 3′SLN and 6′SLN respectively correspond to Neu5Acα2–3 and Neu5Acα2–6 linked to LN. The biotinylated glycans were obtained from the Consortium of Functional Glycomics through their resource request program. Streptavidin-coated High Binding Capacity 384-well plates (Pierce) were loaded to the full capacity of each well by incubating the well with 50 µl of 2.4 µM of biotinylated glycans overnight at 4°C. Excess glycans were removed through extensive washing with PBS.

The trimeric HA unit comprises of three HA monomers (and hence three RBS, one for each monomer). The spatial arrangement of the biotinylated glycans in the wells of the streptavidin plate array favors binding to only one of the three HA monomers in the trimeric HA unit. Therefore in order to specifically enhance the multivalency in the HA-glycan interactions, the recombinant HA proteins were pre-complexed with the primary and secondary antibodies in the ratio of 4∶2∶1 (HA:primary:secondary). The identical arrangement of 4 trimeric HA units in the precomplex for all the HAs permits comparison between their glycan binding affinities.

A stock solution containing appropriate amounts of Histidine tagged HA protein, primary antibody (Mouse anti 6X His tag IgG) and secondary antibody (HRP conjugated goat anti Mouse IgG (Santacruz Biotechnology) in the ratio 4∶2∶1 and incubated on ice for 20 min. Appropriate amounts of precomplexed stock HA were diluted to 250 µl with 1% BSA in PBS. 50 µl of this precomplexed HA was added to each of the glycan-coated wells and incubated at room temperature for 2 hrs followed by the above wash steps. The binding signal was determined based on HRP activity using Amplex Red Peroxidase Assay (Invitrogen, CA) according to the manufacturer's instructions. The experiments were done in triplicate. Minimal binding signals were observed in the negative controls including binding of precomplexed unit to wells without glycans and binding of the antibodies alone to the wells with glycans. The binding parameters, cooperativity (*n*) and apparent binding constant (*K_d_'*), for H2 HA-glycan binding were calculated by fitting the average signal value (from the triplicate analysis) and the HA concentration to the linearized form of the Hill equation: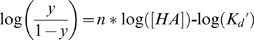, where y is the fractional saturation (average binding signal/maximum observed binding signal). The theoretical *y* values calculated using the Hill equation 
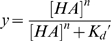
 (for the set of *n* and *K_d_'* parameters) were plotted against the varying concentration of HA to obtain the binding curves for representative human (6′SLN-LN) and avian receptors (3′SLN-LN) shown in **Figure 6**.

### Human respiratory tissue binding assay

Formalin fixed and paraffin embedded normal human tracheal and alveolar tissue sections were purchased from US Biological and US Biomax, respectively. Tissue sections were incubated for 30 minutes in a hybridization oven at 60°C to melt the paraffin. Excess paraffin was removed by multiple washes in xlyene. Sections were subsequently rehydrated in a series of ethanol washes. In order to prevent nonspecific binding, sections were pre-blocked with 1% BSA in PBS for 30 minutes at room temperature (RT). For the generation of HA-antibody precomplexes, the histidine tagged purified recombinant HAs (*Alb58*, *CkPA04*, LS and TLS) were incubated with primary antibody against his tag (mouse anti 6X His tag, Abcam) and secondary (Alexa Fluor 488 goat anti mouse IgG, Invitrogen) antibody in a ratio of 4∶2∶1 respectively for 20 minutes on ice. Tissue sections were incubated with the HA-antibody precomplexed unit, diluted to different final concentrations in 1%BSA-PBS, for 3 hours at RT. Sections were then incubated with propidium iodide to counterstain the nuclei (Invitrogen; 1∶100 in TBST) for 20 minutes at RT. After thorough washing, sections were mounted and analyzed using a Zeiss LSM510 laser scanning confocal microscope.
